# Mimicry and affective disorders

**DOI:** 10.3389/fpsyt.2022.1105503

**Published:** 2023-01-25

**Authors:** Maike Salazar Kämpf, Philipp Kanske

**Affiliations:** Clinical Psychology and Behavioral Neuroscience, Institute of Clinical Psychology and Psychotherapy, Technische Universität Dresden, Dresden, Germany

**Keywords:** mimicry, affective disorders, depression, bipolar disorders, social interactions, manic and depressive episodes

## Abstract

Mimicry, the spontaneous imitation of an interaction partner, is an important part of everyday communication, as it has been shown to foster relationships and increase closeness. People with affective disorders often have problems in their interpersonal lives. In this review, we pursue the question if these problems are linked to differences in mimicry behavior. First, we summarize existing evidence on mimicry, depression and mood. Then, based on five theories differing in their core assumptions regarding mechanisms and functionality of mimicry, we derive suggestions on how mimicry might affect people suffering from bipolar disorders, dysthymia or depression. Depending on each theory, a different understanding of affective disorders and mimicry arises, and we show how the evidence fit in with the suggested theories. Previous studies on affective disorders have focused on mimicry behavior of participants watching photos, computer-generated images, or short video sequences. This review sheds light on the fact that evidence on mimicry needs to be broadened systematically for people with affective disorders in interactional settings. Mimicry represents a novel and important yet underestimated source for diagnostic, intervention and evaluation processes in affective disorders.

## 1. Introduction

Mimicry, the spontaneous imitation of an interaction partner, can occur in different ways. These include imitating verbally—such as using similar words, accent echoing, intonation or speech rate—and non-verbally, like mirroring facial expressions, postures or gestures (1). Mimicry fosters relationships (2, 3), smoothes interactions and increases closeness (4). It is further connected to affective empathy (5) and part of our everyday social communication (6). When mimicry is hindered or disturbed, it can impair emotion recognition (7) and lead to elevated stress reactions of the interaction partner (8). In sum, mimicry plays an important role in social interactions.

Social contacts and companionship are a central part of our life and we engage with others on a daily basis (9). Therefore, it is not surprising, that difficulties in social interactions are associated with mental disorders (10, 11). This is especially true for affective disorders, as they are inherently linked to impairments in social competence (12). Hence, theoretical approaches have been considering the entwinement of affective disorders and problems in interpersonal relationships for decades [e.g., (13–15)]. When referring to social deficits that are associated with affective disorders, we mean “impairments in the subject’s capacity to integrate behavioral, cognitive, and affective skills to flexibly adapt to diverse social contexts and demands, resulting in behavioral outcomes which are judged as negative according to the standards of the specific social context.” [(16), p. 11]. In this review, we pursue the question if the problems people with affective disorders suffer from in their interpersonal lives are linked to differences in mimicry.

Interestingly, it has been found that in depressive states mimicry is decreased (17–19), whereas in positive mood mimicry is increased (19). People with mental illnesses often show fewer facial expressions (20), lower levels of mimicry than psychotherapists during sessions (21) and sometimes atypical facial reactions to emotional expressions of others (22). Moreover, symptom severity of depression is associated with patients showing fewer affiliative and more non-affiliative facial expressions (23). This suggests that people with affective disorders might show less (during depressive episodes) or atypical (during manic episodes) mimicry behavior, which may in turn influence their social relationships. Astoundingly, despite the clinical relevance of understanding the mechanisms underlying mimicry in regards to affective disorders, studies on mimicry in clinical populations are sparse (21).

In this short review we will give an overview on mimicry and affective disorders, namely, major depression, bipolar disorders and dysthymia, in accordance with the DSM-5. We first summarize the existing research on mood and depression, as to the best of our knowledge bipolar disorders and manic states have not been investigated in regard to mimicry. Afterward we present five theoretical approaches to mimicry, from which we will derive suggestions on how mimicry might affect affective disorders. At the end of this review, we will suggest possible future research on mimicry and affective disorders.

## 2. Mimicry and affective disorders

As the evidence on mimicry and affective disorders is sparse, we also included evidence on mood in non-clinical populations.

### 2.1. Studies on mood and mimicry

Different studies indicate that the extent to which we mimic is affected by our emotional state: Fear (24) and positive mood (19) seem to increase our mimicking behavior; sad mood seems to decrease mimicry (18). Moody et al. (24) found that fear induction via audio and film clips increased fear expressions to angry and fearful faces, whereas responses to neutral faces remained unchanged. A small positive correlation was found between self-reported mood and mimicry. Participants who watched a positive film mimicked the person in the video, whereas the group who watched the negative film did not (19). When inducing two different kinds of mood (with happy and sad film clips) participants in a sad mood generally showed less facial mimicry than subjects in a happy mood (18).

All three studies used electromyography to measure facial muscle reactions toward picture or video stimuli. It is unclear if the results would be replicated in a clinical population and in a more naturalistic setting, like real dynamic social interactions. In sum, mood seems to affect how much we mimic others; however, little is known about how much we are being mimicked depending on our mood. In the following we will summarize the evidence on depression and mimicry.

### 2.2. Studies on depression and mimicry

Studies have shown that sadness increases self-focused attention, hence one could assume that people with depression might have greater difficulties to direct their attention toward external social stimuli (18) and therefore show less mimicry behavior and also be less receptive to being mimicked. This might contribute to a further understanding of the fact that people with depression often have poorer social competence (12). Furthermore, people with depression often have the expectation that they might be rejected or that it is too exhausting to engage with others (25, 26), which, may also lead to less mimicry behavior.

Paz et al. (27) even state that depression might be contagious and spread through mimicry. They refer to studies, where the probability of becoming discontent or depressed increases when being in contact with discontent or depressed close others [e.g., (28)]. The authors suggest that the mechanism behind depression spreading in this way are social interactions. During those interactions mimicry activates afferent feedback and consequently emotional contagion takes place. However, they also state that the closeness of the relationship between interaction partners and the mood that they are in can influence the amount of mimicry. Moreover, they add that most studies tested reaction toward pictures or video stimuli and not actual interactions. Few studies have investigated mimicry and depression and the methods on how mimicry is assessed—similar to the studies on mood—mostly include reactions measured by facial electromyography toward static pictures of different emotional expressions and short emotional videos. Here we summarized all studies we are aware of:

Patients with depression showed less mimicry of pictures of happy and sad faces compared to a non-clinical control group (29). However, the 28 patients and 28 non-clinical participants only viewed 30 static pictures and comparison of the two groups did not yield a significant result.

Subclinical dysphoric participants did not show any differences compared to non-dysphoric participants in their facial mimicry reactions in response to pictures of sad faces (30). Dysphoric participants mimicked happy faces less than the non-dysphoric group. The authors did not find differences in emotion recognition between both groups. Yet, they only measured the facial reaction to static pictures.

Acutely depressed patients compared to remitted patients and non-clinical participants showed less mimicry of happy faces and were also less accurate in recognizing happy faces, yet reduced mimicry did not mediate these deficits (31). Moreover, no differences in mimicry of sad faces were found. Furthermore, acutely depressed participants were less confident in their judgment of happy and angry faces and reported difficulties to recognize happy, angry and fearful faces compared to the non-clinical group. Remitted depressed patients were less confident recognizing anger and found it more difficult to recognize happy, angry and fearful faces than the non-clinical group; however, they did not show differences in electromyography parameters. The authors state that if the stimulus material would have been more difficult to decode, differences between the groups might have been more pronounced. They suggest that mimicry affects mostly the speed of emotion recognition and might be part of decoding processes of more ambiguous or complex facial expressions.

The imitation of patients with remitted depression by their interviewers was found to be related to a reduced risk of recurrence of depression and higher participants’ satisfaction with the interaction (32). This might point toward possible positive effects for people with depression being mimicked. To gain a better understanding of the presented results we will now present different theoretical approaches to mimicry.

## 3. Theoretical approaches to mimicry

Processes that underlie interpersonal relationships include social learning, empathy, affiliation and the detection and processing of social stimuli (16). The following theories are to our knowledge the five most important approaches for explaining why mimicry might play such an important role for our relationships. Each one of the theories referring to one of these social processes and therefore leading to divergent assumptions on what the role of mimicry might be for affective disorders (for an overview see Table 1). Importantly, some of the approaches suggest different mimicry behavior depending if a person is experiencing a manic or a depressive episode. We therefore derive different suggestions from these theories for mimicry and depression, dysthymia, mania and hypomania.

**TABLE 1:** Theoretical overview on mimicry and affective disorders.

Theory	Process and function of mimicry	Mimicry and dysthymia	Mimicry and depressive episodes	Mimicry and manic episodes	Mimicry and hypomanic episodes
Social learning theory (33)	Social learning: Attention, retention, reproduction and motivation influence mimicry behavior	Disturbed attention toward negative information might lead to enhanced mimicry of negative emotions, problems with retention and motivation to less mimicry in general		Process positive information faster and might therefore mimic positive emotions more	No differences have been found compared to healthy controls
Perception-action-coupling (38)	Social understanding and empathy: Simulating the observed behavior of others leads to an inner representation of that behavior by activating the same neural pathways, which then facilitates to emotional contagion, mutual understanding and affective empathy	No research to our knowledge	Inconclusive research	Increased affective empathy in bipolar patients during manic episode	No differences
Embodiment theory (41)	Social perception (detection and processing of social stimuli): Cognition is grounded in perceptual, somatosensory and motor experiences. The embodied simulation helps for example emotion recognition performance.	Depending on their mimicry behavior one should find differences in emotion recognition performance			
Social glue approach (2)	Social affiliation: Mimicry acts as a social glue and the desire to affiliate should influences the amount of mimicry shown	Social withdrawal, in other words with lower levels of desire to affiliate, people should be linked to lower levels of mimicry in general (regardless of valence or context)		A heightened desire to affiliate should be linked to elevated levels of mimicry in general (regardless of valence or context)	
Social regulator theory (6, 47)	Social affiliation: Mimicry is (a) a goal-dependent (b) carries meaning (affiliation), and is (c) context-dependent	For example, people with depression, as they show very little or sad facial expression, should be mimicked less and as they often withdraw socially, they should also mimic others less.		Depending on the context: Higher levels of mimicry for affiliative emotions and a positive effect where this mimicry fits the context. However, unfitting or abnormal mimicry behavior would mean that unusual social signals are sent to others which in turn might lead to misunderstandings in interpersonal contexts.	

### 3.1. Social learning theory

The *social learning theory* (33) suggests that observing and imitating others is central for learning. This learning process occurs through attention (only the behaviors that grab our attention can be imitated), retention (we need to be able to have our own inner representation of the behavior we imitate), reproduction (we can only reproduce what we are physically able to reproduce), and motivation (we only reproduce what we think is important enough). Accordingly, we should see differences in mimicry for people with affective disorders. As people suffering from a depressive episode show enhanced attention toward negative information (34), problems with retention, i.e., cognitive deficits in executive function and memory (35), and motivation (36), one could hypothesize that enhanced attention toward negative information might lead to elevated levels of mimicry of negative emotions, whereas problems with retention and motivation may lead to less mimicry in general. People suffering from a manic episode process positive information faster (37) and might therefore mimic positive emotions more. Interventions that tackle these processes should consequently also affect mimicry behavior.

### 3.2. Perception-action-coupling

The *theory on perception-action-coupling* (38) claims that simulating the observed behavior of others leads to an inner representation of that behavior. This inner representation automatically facilitates that same behavior in us; hence we mimic others’ behaviors: Through witnessing others’ emotions, we activate the same neural pathways as if experiencing the emotions ourselves, which then facilitates emotional contagion, mutual understanding, and affective empathy (5, 22). Patients with affective disorders might show interindividual differences in empathy, depending on whether they mimic more or less. In this sense, mimicry might be a tool to regulate empathic responses. If people with affective disorders show inappropriate levels of social mimicry due to altered coupling between perception and action these might contribute to interpersonal problems. It would be interesting to know if this is due to problems in perceiving social cues and/or problems in translating it into mimicry and how this affects patients’ social lives. For depressive episodes, empathy research is inconclusive; while some suggest higher levels of affective empathy (39), other research suggests no differences compared to healthy controls (40), which makes it difficult to draw clear conclusions for mimicry regarding the theory of perception-action-coupling. As people have shown increased levels of affective empathy during a manic episode (40) one could hypothesize higher levels of mimicry. Research on mimicry might shed some light on the relationship between affective disorders and empathy.

### 3.3. Embodiment theory

Another theoretical approach to explain mimicry behavior is the *embodiment theory* (41), which proposes that cognition is grounded in perceptual, somatosensory and motor experiences. The embodied simulation represents the connection between the sensorimotor and cognitive system. Arnold and Winkielman (2) refer to studies where participants are asked to inhibit their own facial expression, which impairs their emotion recognition performance. Further, lesions and temporary inactivation of sensory-motor areas can impair emotion recognition [e.g., (42)]. Zwick and Wolkenstein (31) claim that mimicry is especially useful in recognition of ambiguous, complex or brief emotions and for an acceleration of the facial emotion recognition process. For individuals with affective disorders, the interpretation of social signals and the identification of ambiguous facial expressions could differ (they could be slower of faster) depending on their mimicry behavior. The first step in order to examine this theory would be to test whether differences in mimicry exist during unipolar depression and different episodes of bipolar disorders and whether these are connected to the processing of emotions. Differences in neuropsychological functioning have been found between people affected by bipolar disorders and unipolar depression. For example, people with bipolar disorder have impaired sustained attention even after recovering from acute episodes (43). In this vein, one could hypothesize that people suffering from bipolar disorder show less mimicry in general compared to non-clinical populations and that this affects their emotion recognition processes.

### 3.4. Social glue approach

Lakin et al. (44) assume that mimicry acts as a *social glue*. The desire to affiliate should influence the amount of mimicry shown (2, 44, 45). During manic episodes, where people may have a heightened desire to affiliate (46) people might show elevated levels of mimicry behavior. Whereas during a depressive episode—which is known to be associated with social withdrawal (16) or in other words with lower levels of desire to affiliate—people should show lower levels of mimicry. Inappropriate levels of mimicry might in turn contribute to problems in their interpersonal lives.

### 3.5. Social regulator theory

Hess and Fischer (6, 47) suggest that mimicry functions as *social regulator*. The authors embed mimicry in a social context with three key assumptions: (a) a goal-dependency of emotional mimicry (the intention to affiliate), (b) the meaning of emotional signals (behaviors that carry affiliation signals such as smiling are more likely to be imitated), and (c) context-dependency of the behavior (e.g., smiling in socially inappropriate contexts reduces mimicry). If the emotion that is mimicked does not fit the context, the authors argue that this is not mimicry but rather a mere reaction to an emotional expression. If the context allows for affiliation, one could hypothesize higher mimicry and positive effects for people during a manic phase, as manic phases can be associated with hypersexuality and a higher desire to affiliate (48). However, abnormal mimicry behavior during both (hypo) manic and depressive episodes would mean that unusual social signals are sent to others which in turn might lead to misunderstandings in interpersonal contexts. For example, as people with depression show rather attenuated or sad facial expressions (23), they should be mimicked less. Also, as they often withdraw socially (16), they should also mimic others less. Now that we have gained an overview about the existing theories and their potential consequences, we will discuss how the results on mimicry, mood and affective disorders fit in with those theories and suggest future research.

## 4. Discussion and future directions

Mimicry is a core process of social interaction and understanding mimicry gives insight into the function and dysfunction of social cognition and behavior (41). There is great potential to incorporate mimicry into current theories, research and therapy of affective disorders. We presented the evidence on mimicry and affective disorders and five mimicry theories from which different conclusions on how and why mimicry is important for people suffering from affective disorders can be drawn. We will now set the evidence in the context of these theories.

Future research could use the approach of the social learning theory (33) and test if attentional processes (34, 37), problems with retention, i.e., cognitive deficits in executive function and memory (35), and motivation (36) in depressive or manic episodes differently affect mimicry behavior and consequently social learning.

The theory of perception-action-coupling (38) suggests through perceiving others behavior, we activate similar neural pathways as if experiencing this behavior or feeling ourselves, which consequently facilitates emotional contagion, and cognitive and affective empathy. In line with this assumption, Vicaria and Dickens (38) suggest that facial expressions should be perceived and processed faster, when the expressions signal danger. Mimicry is indeed influenced by fearful mood in non-clinical participants (24): fearful faces are mimicked more, however, angry faces are not mimicked instead participants react with fearful expressions. Paz et al. (27) propose that depression might spread through mimicry by activating afferent feedback and thereupon emotional contagion, which is in line with the core assumptions of the perception-action-coupling theory. Therefore, the relationship between emotional contagion, empathy and mimicry should be evaluated in regard to affective disorders in a dyadic setting, as until now only reactions toward pictures and videos have been studied.

According to the embodiment approach (41) disturbed cognitive processes should be connected to disturbances in social mimicry. Even though the processing of social stimuli is crucial for successful interactions (e.g., to rapidly identify if the facial expression indicates friend or foe) the processing of social stimuli in affective disorders need further investigation (16). To our knowledge studies investigating affective disorders and mimicry have not focused on ambiguous facial or attentional processes yet. It would be interesting to examine if problems in social interactions might be due to disturbed inner representations of social signals, which lead to cognitive distortions, dysfunctional beliefs, and information-processing biases that are typical for people with affective disorders (49). It would be further fascinating to know if the hyperactive amygdala and hypoactive prefrontal regions typical for depression (49), as well as other biological markers like heart rate and skin conductance, are associated with altered mimicry in affective disorders.

In line with the social glue approach (44) mimicry should be especially relevant for creating relationships. Following the core assumptions of the social glue approach, people with acute depression withdraw socially (16, 23) and show lower levels of mimicry of pictures (31). However, studying mimicry in social interactions is still needed. As there are no studies investigating the social effects of mimicry in people with affective disorders, we can only hope that future research will dedicate more time and effort toward this topic. For example, by examining if patients with affective disorders can improve their social relationships through mimicry.

The social regulator theory (6) states that non-affiliative expressions like anger, disgust and excessive sadness should be mimicked less. In line with this assumption Fischer et al. (50) found that disgust is not mimicked, however the assumption is contradicted by a study by (24) that found that fear increases fear expressions to angry and fearful faces, as neither anger nor fear are “affiliative” emotions. However, most of the studies on psychological disorders recorded facial reactions towards emotional pictures and video stimuli. Interestingly, subclinical depressed patients and acutely depressed patients did not show differences in the mimicry of sad faces, however, they mimicked happy faces less (30, 31). According to the social regulator theory (6), this means that they show less affiliative signals, and this might impact their social relationships.

The assumption that these affiliative signals affect social relationships could be fruitful for future research. In line with the social glue approach (44) and the social regulator theory (6) reduced mimicry might be interpreted by others as a lack of affiliative tendencies, and thus lead to reduced liking (3, 18, 51), and consequently to avoidance of people with depression by others (13). This would point toward a vicious cycle with negative mood enhancing social exclusion and vice versa (15, 18). It would be fascinating to know how others react toward these patterns, as for now, most of the results are drawn from non-verbal reactions of patients watching video or picture stimuli and not interacting with others. To our knowledge, studies on verbal mimicry and affective disorders are still missing.

Moreover, social settings require different behaviors: social norms and scripts influence our behavior, which means that most mimicry studies are impoverished compared to natural settings (45). In laboratory settings, stimulation is reduced compared to natural settings, like the elimination of background noise, visual and other stimulation. Experimental paradigms that take place in isolation are not suited to investigating social interactions, as social interactions are inherently dynamic patterns between different agents (52, 53). The results obtained by studies with participants passively watching photos, computer-generated images or short video sequences on a computer screen, are not the same as a naturalistic interaction, nor is copying finger movements or interacting with a confederate or virtual agent. Social interactions are dynamic interactional processes that require online flexibility and adjustments (52, 54, 55) and it is unclear how the results of these studies are transferable. When comparing different experimental approaches one can find “profound differences in behavioral and neural measures during actual social interactions, as compared to engaging participants as mere observers” (p. 1)–especially when looking at people with mental disorders (56). Repetitive inappropriate social behaviors, which often results in a progressive withdrawal from social living and in turn contribute to further worsening of symptoms (16), become obvious in real life interactions and not in passive experimental approaches. Studying mimicry (an inherently interactional phenomenon) in a real-life environment hence represents an important yet underestimated source for clinical research and practice in affective disorders.

## Author contributions

MS and PK contributed to the conception of the review. MS wrote the first draft of the manuscript. PK provided critical feedback and added sections to the manuscript. Both authors contributed to manuscript revision, read, and approved the submitted version.
